# Diagnostic and therapeutic challenges in claudin 18.2-positive gastric cancer treated with zolbetuximab: Intrapatient heterogeneity or secondary loss of expression?

**DOI:** 10.1007/s00432-026-06447-3

**Published:** 2026-03-19

**Authors:** O. Morath, U. Lindig, K. Katenkamp, N. Gassler, D. Haziri, V. Auletta, D. Bauerschlag, T. Franiel, M. Bergener, A. S. Griessbach, T. Ernst, A. Hochhaus, C. C. Crodel

**Affiliations:** 1 Abteilung Hämatologie Und Internistische Onkologie, Klinik Für Innere Medizin II, Universitätsklinikum Jena, Am Klinikum 1, 07747 Jena, Germany; 2 Institut Für Rechtsmedizin, Sektion Pathologie, Universitätsklinikum Jena, Jena, Germany; 3 Klinik Für Innere Medizin IV, Universitätsklinikum Jena, Jena, Germany; 4 Klinik Und Poliklinik Für Frauenheilkunde Und Fortpflanzungsmedizin, Universitätsklinikum Jena, Jena, Germany; 5 Institut Für Diagnostische Und Interventionelle Radiologie, Universitätsklinikum Jena, Jena, Germany

**Keywords:** Claudin-18.2, Biomarker, Gastric cancer, Zolbetuximab

## Abstract

**Background:**

Claudin 18.2 (CLDN18.2) has emerged as a promising therapeutic target in advanced gastric cancer (GC). Data on resistance to zolbetuximab due to secondary antigen loss or baseline intrapatient heterogeneity are sparse.

**Case presentation:**

A young female patient with advanced GC and severe anemia and thrombocytopenia due to bone marrow carcinomatosis, treated with zolbetuximab-based therapy, exhibited discordant CLDN18.2 expression between primary and metastatic sites. Histological analysis of the resected Krukenberg metastasis revealed CLDN18.2 negativity, contrasting with the strong, diffuse expression in the primary tumor and bone marrow metastases. Subsequent disease progression occurred predominantly in lymph node metastases.

**Discussion:**

This case reveals the potential clinical impact of the heterogeneity of CLDN18.2 expression or secondary loss of antigen on the efficacy of CLDN18.2-targeted therapy. Reassessment of biomarkers at progression could be considered to optimize personalized treatment strategies. Furthermore, this case supports the safety of administering zolbetuximab-based therapy in the setting of bone marrow carcinomatosis, even in the presence of severe thrombocytopenia (platelet count < 50 × 10^9^/l). To our knowledge, this represents the first documented case of CLDN18.2-positive gastric cancer identified by bone marrow biopsy worldwide.

## Introduction

Gastric cancer (GC) is one of the primary causes of cancer-related deaths worldwide (International Agency for Research on Cancer 2025), and young patients tend to have a poor prognosis (Cheng et al. 2020). Current guidelines for GC and gastroesophageal junction adenocarcinoma (GEJ) recommend assessment of claudin 18.2 (CLDN18.2) expression, HER2 (human epidermal growth factor receptor 2) status, PD-L1 (programmed death-ligand 1) combined positivity score (CPS) and tumor area positivity (TAP), mismatch repair deficiency (dMMR)/microsatellite instability-high (MSI-H) (Lordick et al. 2025; Lordick et al. 2022). These biomarkers are pivotal for first-line treatment decisions.

Through malignant transformation and loss of cellular polarity, CLDN18.2 is exposed on the surface of tumor cells in the gastric mucosa (Sahin et al. 2008). Its expression does not correlate with overall or progression-free survival and is therefore considered a predictive, but not prognostic, biomarker (Kubota et al. 2022; Choi et al. 2024; Dottermusch et al. 2019).

Zolbetuximab, a recombinant, chimeric monoclonal antibody targeting CLDN18.2, has been approved for first-line treatment in combination with fluoropyrimidine (5-FU) and platinum-based chemotherapy in patients with locally advanced, unresectable, or metastatic HER2-negative GC/GEJ (Keam 2024). The efficacy of zolbetuximab has been examined in the phase III trials (SPOTLIGHT, GLOW): in patients with ≥ 75% CLDN18.2 expression, the addition of zolbetuximab to mFOLFOX6 or CAPOX significantly improved OS: 18.2 vs. 15.5 months and 14.4 vs. 12.2 months, respectively (Shitara et al. 2024b; Shah et al. 2023). Nevertheless, several challenges remain in clinical practice when managing patients treated with zolbetuximab.

## Case presentation

A 36-year-old female refugee presented initially in the 18th week of pregnancy with heavy vaginal bleeding, leading to a miscarriage and subsequent curettage in Ukraine. During a routine clinical examination, an enlarged left supraclavicular lymph node was noted, prompting further diagnostic workup. Histological examination of the excised lymph node revealed a metastasis of a poorly differentiated adenocarcinoma.

One month later, the patient was admitted to the emergency department of the Jena University Hospital following an episode of syncope and extensive spontaneous bruising. Laboratory investigations revealed a severe normochromic normocytic anemia with a hemoglobin level of 2.8 mmol/l (normal range 7.6–9.5 mmol/l) and profound thrombocytopenia 6 × 10^9^/l (normal range 150–360 × 10^9^/l). Peripheral blood smear showed a leukoerythroblastic reaction (40 erythroblasts/100 nucleated cells) and a schistocyte count of 17.3‰ (Table 1). During the initial phase of hospitalization, the patient required intensive transfusion support with packed red blood cells and platelets. Vitamin B12 and folate deficiencies were excluded, the Coombs test was negative.

**Table 1:** Initial laboratory parameters

Parameter	Value		Reference range
Cr	49	µmol/l	44–80
DBil	11.2	µmol/l	< 5.0
TBil	32	µmol/l	< 21
CRP	39.8	mg/l	< 5.0
ALT	0.28	µmol/l*s	< 0.58
GLDH	0.154	µmol/l*s	< 0.08
ALP	11.21	µmol/l*s	0.58–1.74
LDH	37.40	µmol/l*s	< 4.20
Ferritin	1296.0	µg/l	13–150
Haptoglobin	< 0.1	g/l	0.3–2.0
WBC	7.3	10^9^/l	4.4–11.3
RBC	1.5	10^12^/l	4.1–5.1
Hb	2.8	mmol/l	7.6–9.5
Hct	0.14	l/l	0.35–0.45
PLT	6	× 10^9^/l	150–360
Schistocytes	17.3	‰	< 0.5
PT	59	%	70–130
aPTT	30.0	s	25.1–36.5
Fibrinogen (Clauss)	1.0	g/l	2.38–4.98

Cr, creatinine; DBil, direct bilirubin; TBil, total bilirubin; CRP, C-reactive protein; ALT, alanine aminotransferase; GLDH, glutamate dehydrogenase; ALP, alkaline phosphatase; LDH, lactate dehydrogenase; WBC, white blood cells; RBC, red blood cells; Hb, hemoglobin; Hct, hematocrit; PLT, platelets; PT, prothrombin time; aPTT, activated partial thromboplastin time

**Fig. 1:**
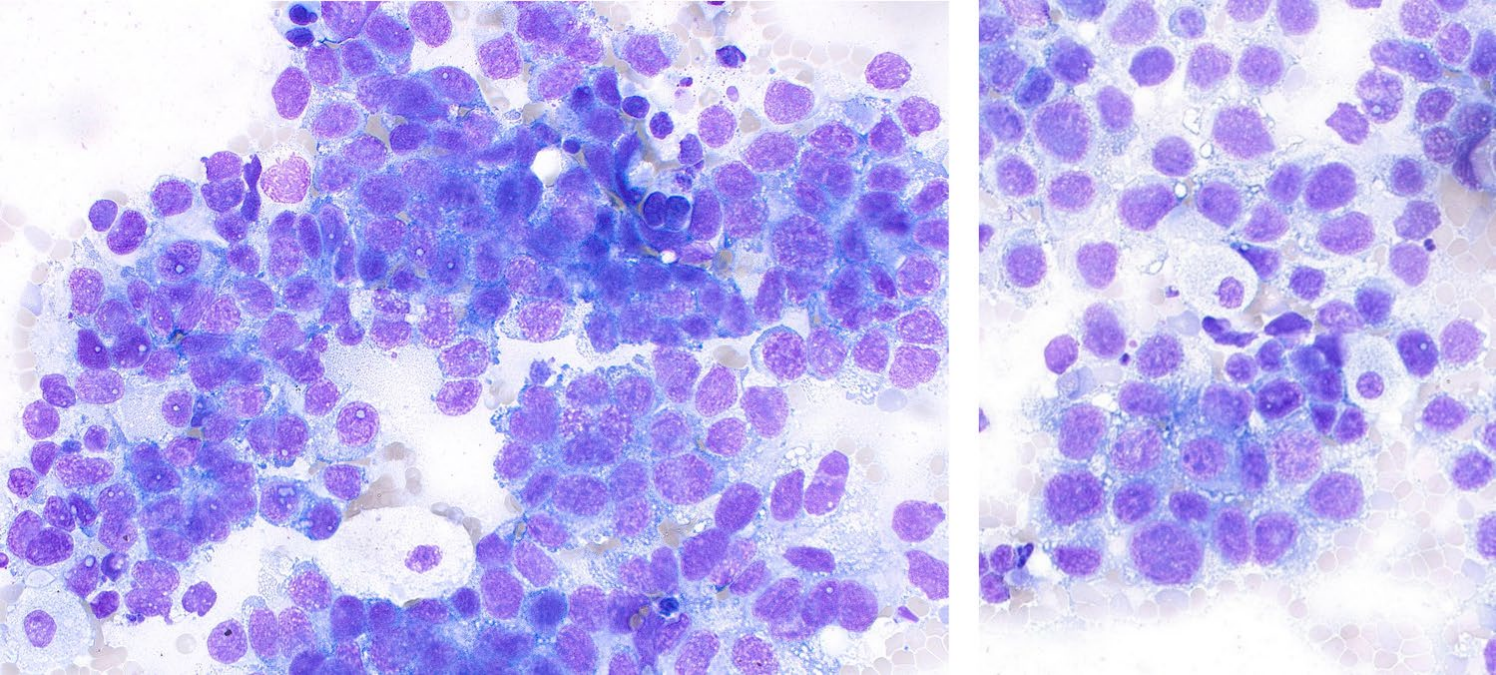
May-Grunwald-Giemsa-stained bone marrow smear showed subtotal bone marrow infiltration with adenocarcinoma cells (× 400)

Initial hematologic findings were suggestive of possible bone marrow infiltration by a non-hematologic malignancy. Cytological (Fig. 1) examination showed subtotal bone marrow infiltration with adenocarcinoma cells. Histological examination revealed subtotal infiltration of the bone marrow by a poorly differentiated adenocarcinoma (grade 3, G3) with predominantly solid growth patterns.

**Fig. 2:**
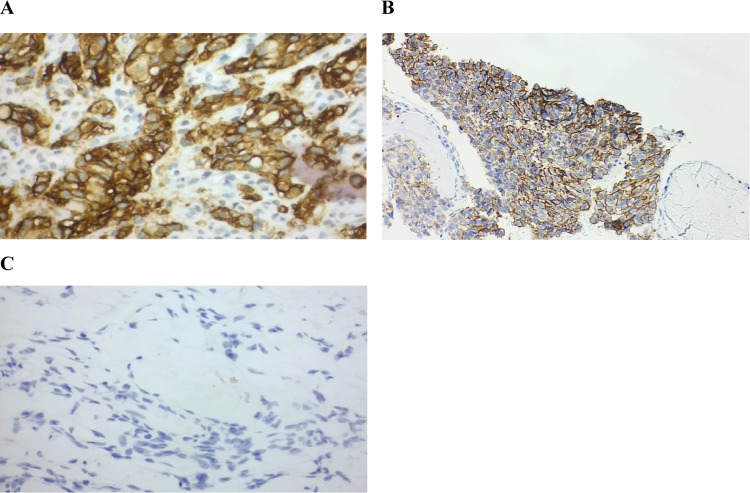
Immunohistochemistry for CLDN18.2 (**A**) Gastric biopsy specimen showing strong membranous CLDN18.2 expression (100%, score 3 +) × 400; (**B**) Bone marrow biopsy showing strong membranous CLDN18.2 expression (100%, score 3 +) × 200; (**C**) Krukenberg metastasis without detectable CLDN18.2 expression (score 0, negative) × 400

A diffusely infiltrating and ulcerating gastric tumor in the corpus has been identified by an upper gastrointestinal endoscopy. Ten representative biopsies were obtained for histological assessment, which confirmed an infiltrating, poorly differentiated adenocarcinoma (G3) with mixed intestinal and diffuse features. Immunohistochemical analysis demonstrated MSS, HER2 score 1 + , PD-L1 TPS: 0%, CPS: 0%, IC: 0%, and CLDN18.2 positive (100%, score 3 +) (Fig. 2A). Immunohistochemical analysis of the bone marrow biopsy has been additionally performed and confirmed strong CLDN18.2 expression (100%, score 3 +) (Fig. 2B).

**Fig. 3:**
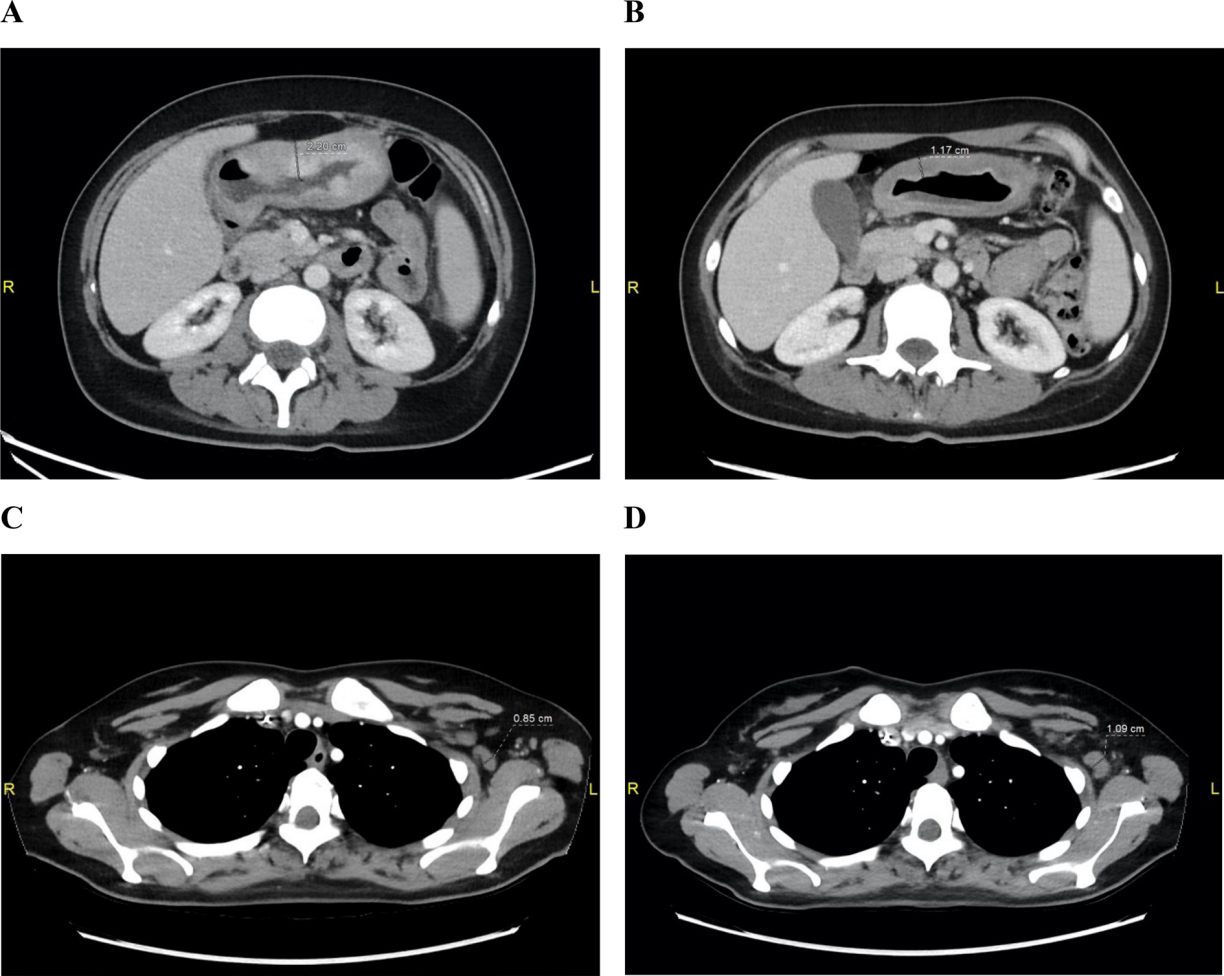
Contrast-enhanced CT imaging at baseline and during systemic treatment (**A**) Baseline CT demonstrating diffuse thickening of the gastric wall in the antrum and pylorus, measuring up to approximately 2.20 cm; (**B**) Follow-up CT performed in January 2025 during systemic treatment with zolbetuximab in combination with oxaliplatin and 5-FU, showing partial remission with regression of gastric wall thickening in the antrum and pylorus to 1.17 cm; (**C**) Follow-up CT performed in January 2025 demonstrating a left axillary lymph node with a short-axis diameter of 0.85 cm; (**D**) Follow-up CT performed in May 2025 during ongoing systemic treatment, showing progressive enlargement of left axillary lymph node metastases with an increase in short-axis diameter to 1.09 cm

CT scans have revealed a diffuse thickening of the gastric wall in the antrum and pylorus, measuring up to approximately 2.20 cm (Fig. 3A), multiple enlarged lymph nodes in the supraclavicular (bilateral), left axillary, mediastinal, right hilar, retrocrural, paraaortic, paracaval, and perigastric regions along the lesser curvature; and a pulmonary nodule in segment 9 of the left lung as well as a micronodule in segment 4 of the right lung, both considered suspicious for metastases. The spine showed disseminated, patchy osteolytic lesions in the thoracic and lumbar vertebrae. Hepatosplenomegaly was present without distinct focal hepatic or splenic lesions. Bilateral ovarian cysts have been observed, measuring up to 40 × 32 mm on the right side. These findings were consistent with T3 N1 M1 GC (UICC stage IV).

**Fig. 4:**
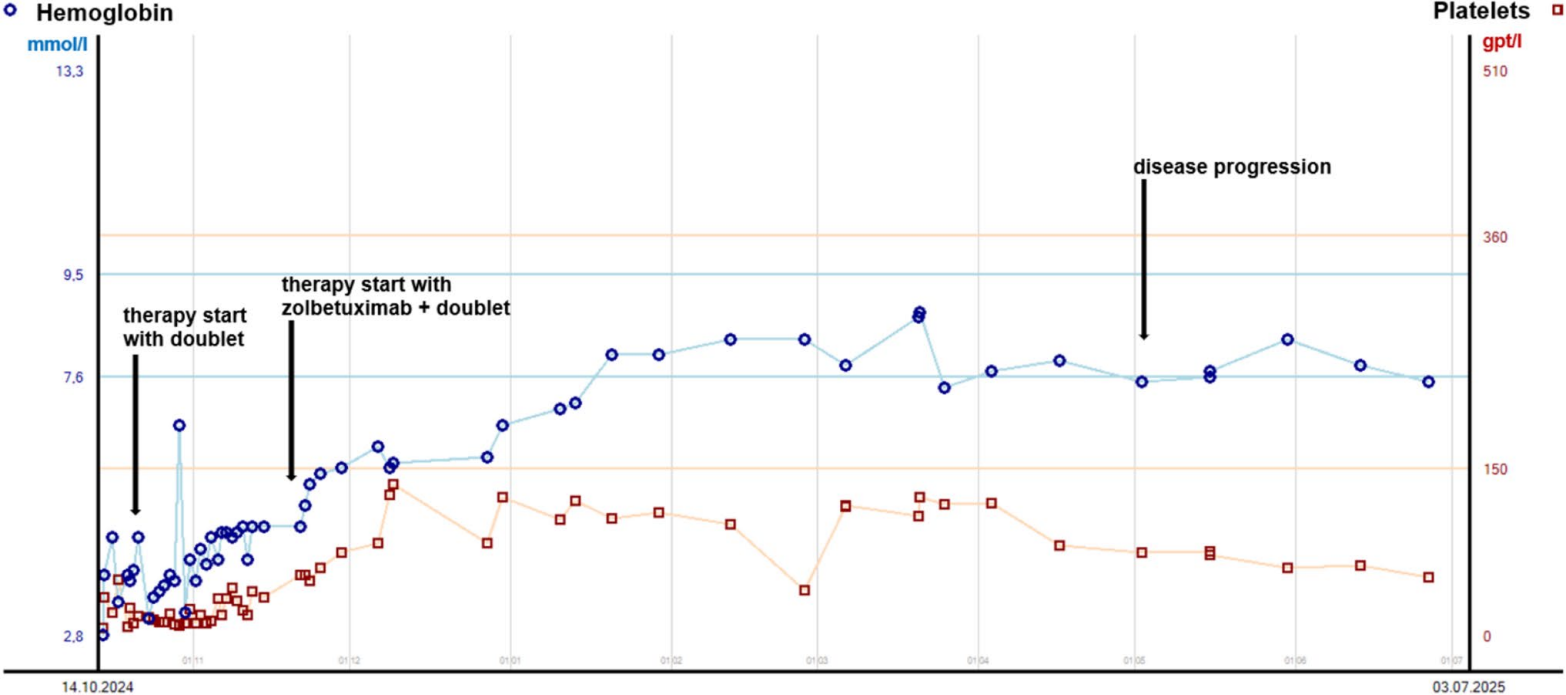
Platelet counts (orange) and hemoglobin levels (blue) before and during systemic therapy *Doublet—oxaliplatin and 5-FU*

Given the profound cytopenias, initial systemic therapy was limited to a reduced-dose combination of oxaliplatin and 5-FU. After placement of a central venous catheter and exclusion of DPD deficiency, systemic therapy has been initiated. With the approval and availability of zolbetuximab in Germany in late 2024, it has been added to oxaliplatin/5-FU. Quadruple antiemetic prophylaxis has been administered, consisting of a 5-HT3 receptor antagonist, an NK1 receptor antagonist, dexamethasone, and olanzapine, in accordance with current guidelines for the prevention of chemotherapy-induced nausea and vomiting in highly emetogenic regimens (Jordan et al. 2021). The patient tolerated the treatment well, with nausea reported as CTCAE (Common Terminology Criteria for Adverse Events) grade 1 throughout the treatment course. Hypoalbuminemia as a potential adverse effect of zolbetuximab was not observed in this case. Under systemic therapy, hemoglobin levels gradually normalized, and platelet counts showed a marked and sustained improvement. This “hematologic recovery” has been correlated with a reduction in transfusion requirements and is suggestive of early treatment response (Fig. 4).

**Fig. 5:**
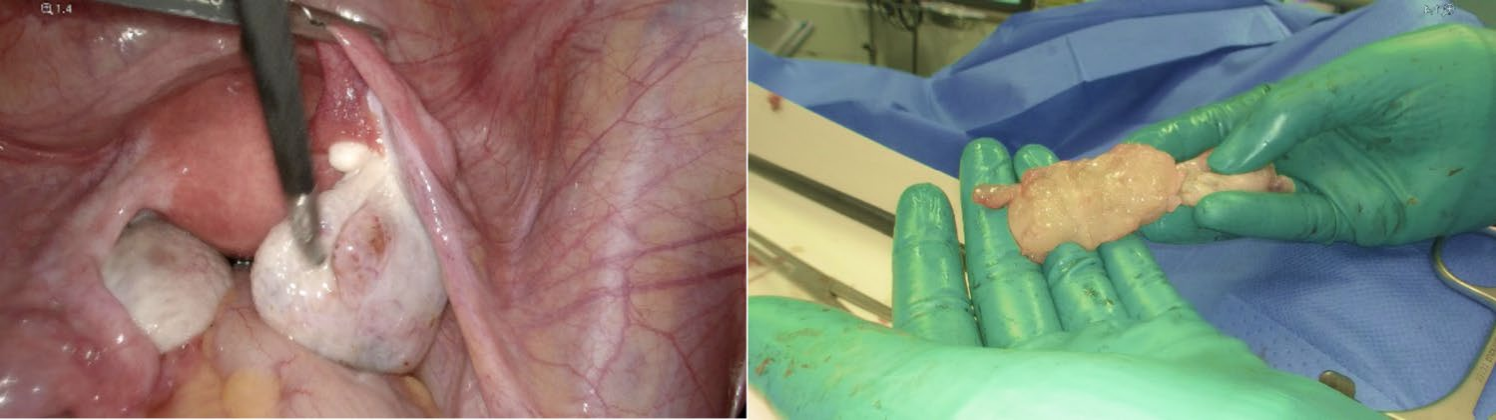
Laparoscopic and extracorporeal image: right ovary with a suspicious drop metastasis

A follow-up CT in January 2025 demonstrated partial remission, with a marked reduction of thoracic and abdominal lymphadenopathy and regression of gastric wall thickening in the antrum and pylorus (Fig. 3B–C). Complete remission has been observed in the previously noted pleural thickening and pulmonary metastasis in segment 8 of the left lung. The mixed osteolytic-osteoblastic bone metastases showed a heterogeneous response under ongoing bisphosphonate therapy, with increasing sclerosis in some lesions and progressive osteolysis in others, indicating a mixed treatment response. A progressively enlarging cystic lesion of the right ovary with solid components raised clinical suspicion for metastatic involvement. In light of these findings, the patient has been referred to gynecological surgery, and laparoscopic adnexectomy was performed (Fig. 5). Histological analysis of the resected ovarian lesion confirmed a Krukenberg metastasis, characterized by poorly differentiated adenocarcinoma. Immunohistochemistry revealed CLDN18.2 negativity (score 0, negative) in the ovarian metastasis (Fig. 2C), in contrast to the strong, diffuse CLDN18.2 positivity in the primary gastric tumor. Notably, laparoscopy was performed approximately 30 days after the last cycle of systemic therapy.

A follow-up CT in May 2025 revealed progressive enlargement of lymph node metastases in the supraclavicular, left axillary (Fig. 3D), mediastinal, upper abdominal, and retroperitoneal regions. A slight but diffuse increase in gastric wall thickening has been observed. The known mixed osteoblastic-osteolytic bone metastases remained stable overall. The patient has been re-presented at the multidisciplinary tumor board. The progression has been classified as interval progression occurring during a temporary pause in systemic therapy, and treatment was continued with the previously initiated regimen of zolbetuximab, oxaliplatin, and 5-FU. A follow-up CT scan performed eight weeks later revealed several newly delineable, pathologically enlarged lymph nodes, confirming overall tumor progression. As a result, a switch to second-line systemic therapy has been initiated. Additionally, an NGS-based panel assay (TruSight Oncology 500-TSO500, Illumina, Inc.) has been performed for genomic profiling of the resected Krukenberg metastasis. The TSO500 analysis did not identify any actionable genomic alterations enabling targeted therapy. However, an oncogenic *TP53* p.Arg282Trp (R282W) mutation has been detected.

## Discussion

This clinical case highlights the complexity of treatment decisions in a young female patient with advanced GC with bone marrow and ovarian metastases. Bone marrow carcinomatosis caused by GC occurs in approximately 1% of cases (Yamamura et al. 1985), often affects younger patients with a female predominance (Xiao et al. 2022; Kwon et al. 2011), and is associated with an extremely poor prognosis (Ergun et al. 2017; Iguchi 2015). Patients typically present with hematological abnormalities such as disseminated intravascular coagulation and microangiopathic hemolytic anemia, which can rapidly worsen clinical outcomes (Zhai et al. 2022). Chemotherapy regimens combining docetaxel, cisplatin, and 5-FU in various combinations have shown potential clinical benefit in this patient group (Ergun et al. 2017; Xiaohui et al. 2022). Efficacy trials of zolbetuximab plus chemotherapy (e.g., SPOTLIGHT, GLOW studies) required platelets ≥ 100 × 10^9^/l; patients with bone marrow carcinomatosis were likely excluded, given the platelet eligibility criteria (Shitara et al. 2024b; Shah et al. 2023). CLDN18.2 is not normally expressed in bone marrow, and to the best of our knowledge, its expression in bone marrow biopsy specimens has not been previously described. Zolbetuximab has been introduced after two prior cycles of oxaliplatin/5-FU, when platelet count was < 50 × 10^9^/L, resulting in a rapid and substantial improvement in hematologic parameters. It remains challenging to differentiate whether the observed “hematologic recovery” was solely due to chemotherapy or partially attributable to the addition of zolbetuximab. The continuous decline in platelet counts, despite dose reductions of oxaliplatin and 5-FU, raised clinical suspicion of disease progression (Fig. 4).

Recent analysis (Choi et al. 2024) demonstrated significant heterogeneity in CLDN18.2 expression between primary and metastatic GC sites. In a cohort of 135 patients, only 25.2% exhibited positive concordance, i.e., CLDN18.2 positivity in both the primary and metastatic tumors, whereas 49.6% showed negative concordance. In the remaining 25.2% of patients, CLDN18.2 expression was discordant—present only in the primary tumor (n = 14) or only in metastases (n = 20). CLDN18.2 expression has been primarily analyzed in metastatic lesions of the peritoneum, liver, and lymph nodes, while rarer metastatic sites such as the ovaries were grouped under “others” (n = 6), underscoring the limited data available on ovarian involvement and the importance of site-specific evaluation. A further study focusing on ovarian metastases originating from GC (Kim et al. 2022) indicated a substantial concordance rate of 92% for CLDN18.2 expression between the primary tumor and ovarian metastases. Only one of 12 CLDN18.2-positive primary tumors in their cohort showed loss of expression in the corresponding Krukenberg metastasis. In the present case, CLDN18.2 expression had not been detected in the ovarian metastasis, despite strong diffuse expression in the initial gastric tumor, indicating an unusual discordance.

Disease progression in the current case mostly appeared in lymph node metastases (Fig. 3D). A study by Rohde et al. (2023) investigated CLDN18.2 expression in 80 lymph node metastases from patients with GC and demonstrated significant variability: in 30% of cases, CLDN18.2 expression in lymph node metastases was lower than in the matched primary tumor, and 16% of cases showed complete loss of expression in at least one metastasis. In the other study (Pak et al. 2025), among 65 paired samples, discordant expression between primary tumors and corresponding lymph nodes was observed in 18.5%. Significantly, CLDN18.2 positivity in the primary tumor frequently diminished in lymph node metastases (positive agreement rate 36.4%), whereas negativity was predominantly preserved (negative agreement rate 90.7%). CLDN18.2-positive tumor clones may not consistently account for corresponding lymph node metastases, or they may lose their CLDN18.2 positivity for reasons that remain unclear (Pak et al. 2025).

Another possible mechanism of resistance to zolbetuximab could be the secondary loss of CLDN18.2, similar to what has been observed for HER2. Loss of HER2 expression is a well-established mechanism of acquired resistance in GC patients treated with trastuzumab-based therapy. Recent studies (Pietrantonio et al. 2016; Seo et al. 2018) demonstrated that loss of HER2 positivity has been observed in approximately 29–32% of patients on post-progression biopsy. Variable expression of PD-L1 between primary tumors and metastases has been shown and may influence the efficacy of immune checkpoint inhibitors as well (Zhou et al. 2020; Massa et al. 2024).

The concept of “rescued” biomarker positivity, known in HER2-positive GC, describes patients who initially test negative for a therapeutic target but are later found to express the target upon repeated biopsy. In the GASTHER-1 study (Seo et al. 2018), additional biopsies have been performed in patients with advanced GC who were initially classified as HER2-negative: 5–8% of these patients were reclassified as HER2-positive, thereby becoming eligible for trastuzumab-based therapy. This change in classification has been largely attributed to intratumoral heterogeneity and sampling limitations during initial testing. Importantly, patients with rescued HER2 positivity showed a less pronounced benefit from trastuzumab (Seo et al. 2018), likely reflecting more heterogeneous or lower-level HER2 expression compared to patients with strong and concordant positivity at baseline. Nonetheless, the study demonstrated that repeat biomarker evaluation could significantly influence treatment decisions, especially in cases of disease progression or unexpected clinical behavior. In the context of the present case, this approach may be relevant to CLDN18.2. Unfortunately, to our knowledge, no comparable clinical data currently exist for rescued CLDN18.2 positivity. With broader use of CLDN18.2-targeted therapy, re-evaluating expression, similar to HER2, may become key for treatment decisions, especially in borderline (CLDN18.2 expression in < 75% of tumor cells) or atypical cases.

Altogether, recent findings indicated the value of CLDN18.2 as a biomarker, but also revealed gaps in the management of patients. It remains uncertain whether all patients with CLDN18.2-positive advanced GC should receive zolbetuximab. First, improvements in OS and PFS in phase III trials (SPOTLIGHT, GLOW) did not translate into higher response rates or longer duration of response, an important consideration in patients with high disease burden or symptomatic metastases (Shitara et al. 2024a, 2024b; Shah et al. 2023). Second, subgroup analyses suggest that patients from non-Asian countries may derive less benefit from zolbetuximab-containing regimens. Third, an unresolved clinical dilemma arises when tumors co-express CLDN18.2 and PD-L1 with a CPS ≥ 1. While a CPS ≥ 10 generally favors immune checkpoint inhibition, the optimal strategy for patients with CPS ≥ 1 but ≤ 10 remains unclear, whether zolbetuximab-based chemotherapy, immune checkpoint inhibition, or a combined approach offers the most effective. Notably, among CLDN18.2-positive tumors, 18–42% show PD-L1 expression with CPS > 5 (Kubota et al. 2022; Pak et al. 2025; Pellino et al. 2021).

In addition to monoclonal antibody therapy, innovative approaches to target CLDN18.2 with antibody–drug conjugates have shown promising early results, with objective response rates of up to 55.6% and disease control rates reaching 88.9% (Xu et al. 2024; Yu et al. 2024). Similarly, in a phase I trial, CLDN18.2-targeted CAR-T cells showed notable clinical efficacy in gastrointestinal cancers, achieving an objective response rate of 48.6% and a disease control rate of 73.0% (Qi et al. 2022).

In conclusion, discordant CLDN18.2 expression between the primary tumor and the ovarian metastasis and subsequent lymphatic progression suggest that tumor heterogeneity or secondary loss may compromise treatment efficacy. The underlying mechanism in this clinical case could not be clearly defined. Nevertheless, these findings emphasize the importance of reassessing of molecular targets during disease evolution. Future research should focus on the genesis of acquired resistance to improve outcomes with CLDN18.2-targeted therapies.

## Data Availability

No datasets were generated or analysed during the current study.
